# Unexplained visual loss in retinal detachment repair: comparing gas, silicone oil and heavy silicone oil by multivariable regression.

**DOI:** 10.1186/s40942-023-00466-9

**Published:** 2023-04-29

**Authors:** George Moussa, Maria Tadros, Soon Wai Ch’ng, Mariantonia Ferrara, Dimitrios Kalogeropoulos, Ash Sharma, Kim Son Lett, Arijit Mitra, Ajai K Tyagi, Walter Andreatta

**Affiliations:** 1 Birmingham and Midland Eye Centre and Academic Unit of Ophthalmology, University of Birmingham, Birmingham, UK; 2 Birmingham Midlands Eye Centre, Sandwell and West Birmingham Hospitals NHS Trust, Dudley Road, Birmingham, B18 7QH UK; 3 Manchester Royal Eye Hospital, Oxford Road, Manchester, M13 9WL UK; 4 Department of Ophthalmology, Faculty of Medicine, School of Health Sciences, University of Ioannina, Ioannina, Greece; 5 Kantonsspital Winterthur, Brauerstrasse 15, 8400 Winterthur, Switzerland; 6 University of Zurich, Rämistrasse 71, 8006 Zurich, Switzerland

**Keywords:** Retinal detachment, Gas, Silicone oil, Heavy silicone oil, Densiron 68, Retina, vitreoretinal, Unexplained visual loss

## Abstract

**Purpose:**

To measure the proportion of unexplained and all causes of visual loss following primary rhegmatogenous-retinal-detachment (RRD) repair, comparing gas tamponade (SF_6_, C_2_F_6_, C_3_F_8_), silicone oil (SO, 1000cs and 5000cs) and heavy silicone oil (Densiron).

**Methods:**

Retrospective, continuous, comparative study from 01/1/2017-31/5/2021. All primary RRDs were included after successful removal of SO and Densiron. Primary failures were excluded. Visual loss was defined as reduction of ≥0.30 logMAR units. Multivariable binary-logistic and linear regression models to compare tamponade, and all cases of unexplained visual loss and logMAR gain were performed. Covariates included age, ocular co-morbidities, pre-op vision, macula-status, high-myopia, giant-retinal-tear (GRT), perfluorocarbon-use, combined buckle/PPV, PVR-C, retinectomy, tamponade agent and post-operative lens status.

**Results:**

Of 1,012 primary RRDs, we found an incidence of unexplained visual loss in 15/1012 (1.5%, SF_6_:1/341[0.3%], C_2_F_6_:4/338[1.2%], C_3_F_8_:2/239[0.8%], Densiron:0/33[0.0%], SO-1000cs:5/43[11.6%] and SO-5000cs:3/18[16.7%]), and visual loss of all causes in 57/1012 (5.6%, SF_6_:13/341[3.8%], C_2_F_6_:14/338[4.1%], C_3_F_8_:15/239[6.3%], Densiron:2/33[6.1%], SO-1000cs:9/43[20.9%] and SO-5000cs:4/18[22.2%]). On multivariable binary-logistic regression, we report that macula-on RRD (Odds-Ratio[OR]5.7,95% Confidence-interval[CI]1.2-28.2, p=0.032), GRT (OR35.0,CI 2.0-617.3, p=0.015), combined buckle/PPV (OR37.7,CI 2.0-711.4, p=0.015), SO1000cs (OR86.6,CI 5.6-1,348.0), p=0.001) and 5000cs (OR37.2,CI 1.3-1,101.5, p=0.036) (Reference-tamponade:SF_6_) were associated with unexplained visual loss. Duration of oil tamponade was not linked to increase in unexplained visual loss (p=0.569).

**Conclusions:**

Correlation between SO in detachment repairs and unexplained visual loss has been established, however incidence with HSO has not been compared to other agents. This study demonstrates that although SO was linked with risk-adjusted increased unexplained visual loss relative to gas tamponade, no such association was found for Densiron, on multivariable analysis.

**Supplementary Information:**

The online version contains supplementary material available at 10.1186/s40942-023-00466-9.

## Introduction

Intraocular tamponade agents represent a fundamental tool in pars plana vitrectomy (PPV) for rhegmatogenous retinal detachment (RRD) repair. Expansile gases compared to silicone oils (SO) and heavy silicone oils (HSO), offer several significant advantages, including the better tamponade effect with spontaneous reabsorption. However, SO and HSO still play a crucial role in the surgical management of RRD with specific characteristics, such as chronicity, posterior and/or multiple retinal breaks, giant retinal tears (GRT), presence of grade C proliferative vitreoretinopathy (PVR), and inability of patients to strictly posture after surgery [1]. Densiron®68 is a heavy SO (HSO) that is a mixture of 30.5% perfluorohexyloctane (F_6_H_8_) and 69.5% SO 5000cs [2] and as such, is intended for inferior tamponade.

Unexplained visual loss is a severe known complication following removal of SO (ROSO) [3, 4] and has been less frequently documented following gas tamponade [3]. However, few studies have compared the incidence of unexplained vision loss between gases and SO [3, 5, 6]. Additionally, studies documenting the incidence of unexplained visual loss and its association with SO, have used “SO” as an umbrella term with no comparison conducted among SO of different viscosities. Finally, only one prior study reported a case of unexplained visual loss after HSO removal [7].

In view of this, in this large sample study we compared the incidence of visual loss, unexplained and of all causes, in eyes with primary RRD treated with PPV based on the tamponade used, such as gases (sulphur hexafluoride [SF_6_], hexafluoroethane [C_2_F_6_] and octafluoropropane [C_3_F_8_]), conventional SO 1000cs and SO 5000cs, and Densiron®68.

## Methods

We conducted a single centre, retrospective, continuous, comparative study on patients that underwent primary RRD at the Birmingham and Midland Eye Centre (BMEC) between January 2017 and May 2021. All the data were extracted from electronic patient records (EPR, Medisoft Ophthalmology, Medisoft Limited, Leeds, UK).

### Inclusion and exclusion criteria

We included all eyes that underwent successful PPV for primary RRD repair. In the cases of SO or HSO tamponade, only eyes that had retina attached after a minimum interval of one-month post-ROSO were included. The eyes were categorised by the tamponade used (SF_6_, C_2_F_6_, C_3_F_8,_ ALCHIMIA Srl, Padova, Italy), conventional SOs 1000cs and 5000cs (FCI silicone oil, France Chirurgie Instrumentation, Paris, France) and HSO (Densiron®68, Fluoron Co, Neu-Ulm, Germany).

To reduce confounding factors, we excluded eyes with primary failure, lack of follow up, tamponade-filling at last review, significant intraoperative complications and aphakia.

#### Surgical procedure

All eyes underwent transconjunctival 23-gauge-PPV with cryotherapy and/or endolaser retinopexy, and gas/SO/HSO tamponade. Combined encircling band, PVR peel, retinectomy and intraoperative use of perfluoro-n-octane (PFCL, Alcon Laboratories Inc, Fort Worth, Texas) were performed if needed. The choice of intraocular tamponade was based on RD findings, patient’s needs and operating surgeon’s preference. Factors favouring the choice of SO/HSO were multiple and/or posterior retinal breaks, the presence of a GRT, presence of PVR Grade C, chronicity of RD, poor ability to posture and only functioning eye. Patients unable to posture and/or with inferior detachments were more likely to receive Densiron®68 compared to SO.

### Outcomes and definitions

Our primary outcome was to compare the proportion of unexplained visual loss following primary RRD repair in the different groups. Secondarily, we assessed the rate of vision loss due to an identifiable cause in each group.

Visual loss was defined as the reduction of visual acuity (VA) by ≥ 0.30 logMAR units from pre-operative assessment to last available post-operative episode. The visual loss was defined as “unexplained” when no identifiable causes could be identified on ophthalmic examination and imaging.

### Data collection

We collected data on baseline demographics (age, gender) and clinical characteristics, (pre-operative lens status, laterality, the presence of high myopia [defined as greater than six dioptres of myopia], pre-operative VA and ocular co-morbidities), baseline RD findings (macula status, PVR grade C, GRT), operative details (tamponade, PFCL use, PVR peel and/or retinectomy, intraoperative complications), and post-operative results (post-operative VA, complications, post-operative lens status, duration of SO/Densiron tamponade).

Two clinicians (GM and MT) reviewed the records of each patient that experienced visual loss to determine whether a clear cause could be identified, including cataract, posterior capsular opacification, vitreous haemorrhage, retinal vascular diseases, advanced age-related macular degeneration, significant postoperative macular oedema, epiretinal membrane, macular hole, and end-stage glaucoma. All patients that were diagnosed with unexplained visual loss had at least an optical-coherence-tomography (OCT) scan showing findings not consistent with the level of visual loss.

### Statistical analysis

All statistical analysis was performed using IBM SPSS Statistics for Windows, Version 28.0 (IBM Corp, Armonk NY). Statistical significance was defined as p < 0.05. By default, for logMAR VA outcomes, preoperative VA and postoperative VA were defined as the better of corrected distance VA (CDVA), uncorrected distance VA (UDVA) or pinhole VA (PHVA) as reported in the national ophthalmology database (NOD) audit [8]. Records in Snellen were converted to logMAR. Low values of VA, corresponding to count fingers (CF), hand movements (HM), perception of light (PL) and no PL (NPL) were substituted with 2.10, 2.40, 2.70 and 3.00 logMAR, respectively, in keeping with previous publications from the NOD group [8], using a tool by Moussa et al. [9].

Prior to analysis, continuous variables were assessed using the Shapiro-Wilk test and found not to be normally distributed. Hence, data are primarily reported as medians and interquartile ranges (IQRs) throughout. For univariate comparisons, Mann Whitney U and Independent-Samples Kruskal-Wallis test were used to compare two and three or more independent groups respectively (age and VA). Wilcoxon signed rank test was used for two-paired VA data. Fisher exact test and Chi-Squared test were used for nominal variables.

Taking into consideration the retrospective design of this analysis and, thus, the significant differences in baseline characteristics between groups, multiple steps have been used to minimise selection bias between groups. All risk factors had univariate analysis for unexplained and all causes of visual loss (Additional file 1: Table S1). However, due to significant differences in baseline case complexity, we undertook multivariable logistic regression analyses to risk adjust between baseline characteristics with unexplained visual loss and all causes of visual loss as dependent variables, including the previously mentioned data collected as independent variables. We risk adjusted for pre-operative logMAR in the regression model as patients with different pre-operative VA will have a different expected visual prognosis [10], further reducing bias in our model.

## Results

**Fig. 1:**
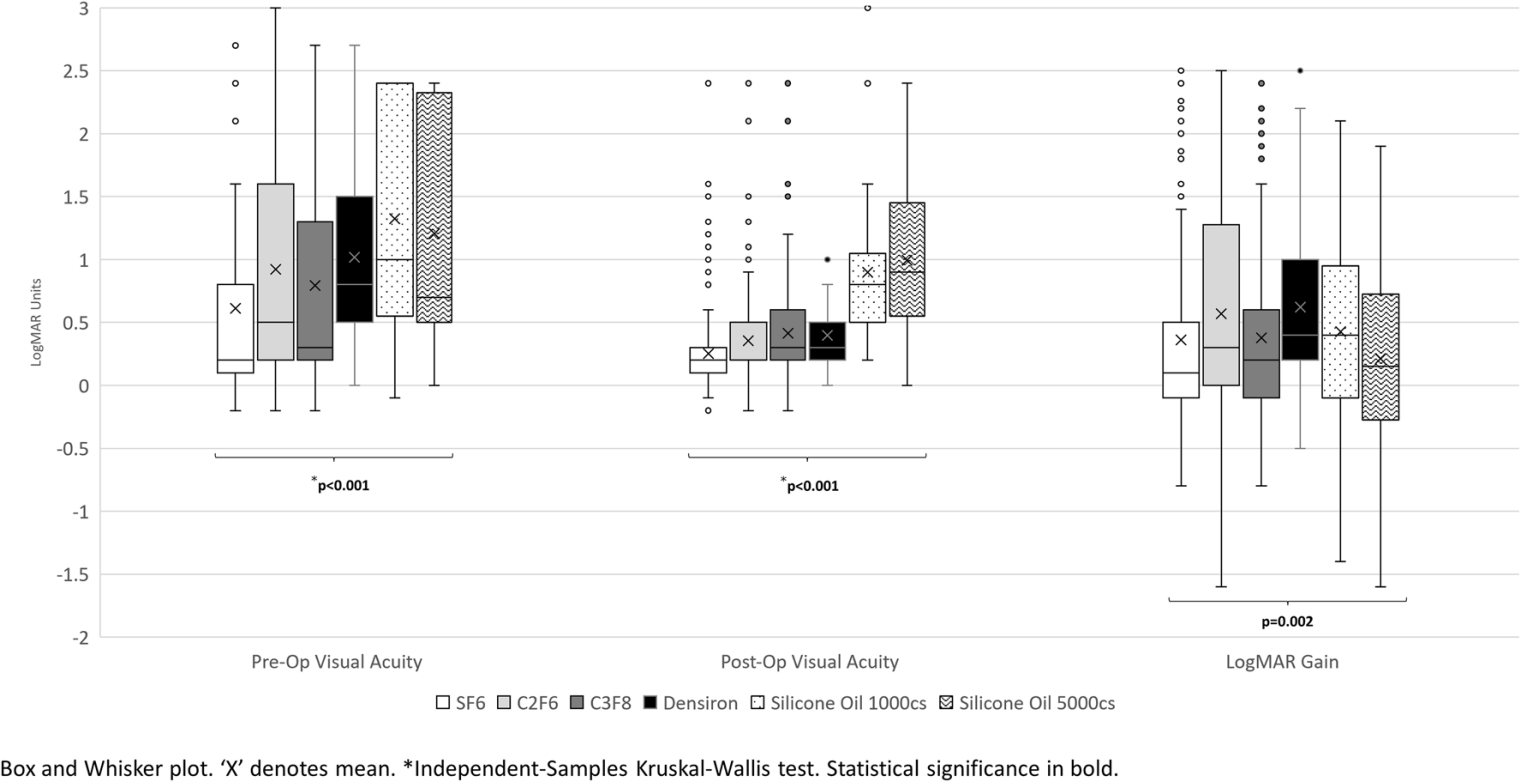
Box and whisker plot of visual acuity baseline and outcomes by tamponade

Of 1053 eyes reviewed, 29 were excluded due to lack of sufficient follow up, and 12 because of aphakia. The remaining 1012 eyes were included in the final analysis, of which 341 received SF_6_, 338 C_2_F_6_, 239 C_3_F_8_, 33 Densiron^®^68, 43 SO 1000cs and 18 SO 5000cs. The baseline characteristics and outcomes of each tamponade is reported in Table 1. As expected, there are several significant differences in baseline characteristics and outcomes of patients with different tamponade agents. Consistently, there are significant differences between pre- and post-operative visual outcomes by tamponade agent (Fig. 1). The rate of unexplained visual loss was significantly different (p < 0.001) in the tamponade-based groups, as it was detected in 7/918 eyes (0.8%) in the gas group (with the minimum rate of 0.3% in SF_6_ group), 0/33 (0.0%) in the Densiron group and 8/61 (13.1%) in the SO group, with no significant difference between 1000cs and SO 5000cs subgroups. The rate of visual loss of known origin also differed significantly among the groups (Table 1). Table 2 summarises all the causes of vision loss identified in each eye.

**Table 1:** Baseline clinical characteristics and outcomes of primary retinal detachments by tamponade agent

	Total	SF_6_	C_2_F_6_	C_3_F_8_	Densiron	SO 1000cs	SO 5000 cs	p Value
Total	1012	341	338	239	33	43	18	–
Age (years, IQR)	59 (53 to 68)	59 (53 to 67)	61 (54 to 67)	59 (52 to 66)	61 (55 to 74)	63 (54 to 71)	55 (37 to 63)	0.087
Gender (% Male)	648 (64.0%)	215 (63.0%)	204 (60.4%)	158 (66.1%)	26 (78.8%)	29 (67.4%)	16 (88.9%)	0.057
Laterality (% Right)	528 (52.2%)	172 (50.4%)	195 (57.7%)	113 (47.3%)	17 (51.5%)	21 (48.8%)	10 (55.6%)	0.213
High Myope (% Yes)	61 (6.0%)	20 (5.9%)	25 (7.4%)	10 (4.2%)	3 (9.1%)	2 (4.7%)	1 (5.6%)	0.659
Ocular Co-morbidities	212 (20.9%)	50 (14.7%)	73 (21.6%)	49 (20.5%)	18 (54.5%)	14 (32.6%)	8 (44.4%)	**< 0.001**
Preoperative Lens								–
Phakic	644 (69.0%)	241 (75.5%)	220 (69.0%)	138 (63.6%)	18 (60.0%)	17 (53.1%)	10 (62.5%)	**0.012**
Pseudophakic	289 (31.0%)	78 (24.5%)	99 (31.0%)	79 (36.4%)	12 (40.0%)	15 (46.9%)	6 (37.5%)	
Macula Status								
Off	497 (49.1%)	116 (34.0%)	192 (56.8%)	122 (51.0%)	23 (69.7%)	32 (74.4%)	12 (66.7%)	**< 0.001**
On	515 (50.9%)	225 (66.0%)	146 (43.2%)	117 (49.0%)	10 (30.3%)	11 (25.6%)	6 (33.3%)	
Giant Retinal Tear	7 (0.7%)	0 (0.0%)	1 (0.3%)	0 (0.0%)	0 (0.0%)	3 (7.0%)	3 (16.7%)	**< 0.001**
PVR C	16 (1.6%)	0 (0.0%)	0 (0.0%)	3 (1.3%)	3 (9.1%)	8 (18.6%)	2 (11.1%)	**< 0.001**
Retinectomy	3 (0.3%)	0 (0.0%)	0 (0.0%)	0 (0.0%)	0 (0.0%)	3 (7.0%)	0 (0.0%)	**< 0.001**
Perfluorocarbon	36 (3.6%)	5 (1.5%)	5 (1.5%)	6 (2.5%)	3 (9.1%)	10 (23.3%)	7 (38.9%)	**< 0.001**
Combined Buckle/PPV	13 (1.3%)	1 (0.3%)	1 (0.3%)	6 (2.5%)	0 (0.0%)	4 (9.3%)	1 (5.6%)	**< 0.001**
Presentation to Surgery (days)	1 (0 to 4)	1 (0 to 1)	1 (0 to 4)	1 (0 to 5)	2 (0 to 7)	2 (0 to 7)	2 (0 to 7)	**< 0.001**
Postoperative Lens								–
Phakic	471 (46.5%)	187 (54.8%)	168 (49.7%)	107 (44.8%)	3 (9.1%)	3 (7.0%)	3 (16.7%)	**< 0.001**
Pseudophakic	541 (53.5%)	154 (45.2%)	170 (50.3%)	132 (55.2%)	30 (90.9%)	40 (93.0%)	15 (83.3%)	
Duration of oil (days)	147 (91 to 215)	–	–	–	84 (68 to 124)	171 (125 to 222)	202 (162 to 308)	**< 0.001**
Visual Loss	57 (5.6%)	13 (3.8%)	14 (4.1%)	15 (6.3%)	2 (6.1%)	9 (20.9%)	4 (22.2%)	**< 0.001**
Unexplained Visual Loss	15 (1.5%)	1 (0.3%)	4 (1.2%)	2 (0.8%)	0 (0.0%)	5 (11.6%)	3 (16.7%)	**< 0.001**

Age and days from review to operation days are reported as median (interquartile range) and Kruskal Wallis test used to compare continuous variables

Chi Squared test to compare more than two nominal groups

Statistical significance in bold

GRT:Giant Retinal Tear, PVR:Proliferative Vitreoretinopathy

**Table 2:** Causes of visual loss accounted for

Patient Number	Tamponade	Macular Status	Pre-Operative Visual Acuity	Post-Operative Visual Acuity	logMAR Gain	Reason for Post-Operative Visual Loss
1	C2F6	On	0.00	0.50	− 0.50	wAMD
2	SF6	On	0.00	0.80	− 0.80	Uveitis
3	C3F8	Off	0.20	0.80	− 0.60	PCO
4	C3F8	Off	2.10	2.40	− 0.30	Decompensated Corneal Graft
5	SF6	On	0.20	0.60	− 0.40	PCO
6	C2F6	On	0.00	0.60	− 0.60	Cataract,ERM,Uveitis
7	C3F8	On	0.00	0.80	− 0.80	Post− operative Macular hole
8	SF6	On	0.20	0.50	− 0.30	Cataract
9	C2F6	On	0.20	0.50	− 0.30	Cataract
10	C3F8	On	0.20	0.60	− 0.40	Cataract
11	C2F6	Off	0.80	1.30	− 0.50	Suprachoroidal Haemorrhage
12	C2F6	On	0.50	1.10	− 0.60	IOL Dislocation
13	C3F8	Off	0.20	0.60	− 0.40	Post-Operative diplopa with occlusion of operated eye at visual check
14	C2F6	On	0.50	2.10	− 1.60	Post-operative submacular haemorrhage following ERM peel
15	C2F6	On	0.20	0.60	− 0.40	Cataract
16	C3F8	On	0.20	2.40	− 2.20	Cataract
17	C3F8	On	0.20	0.60	− 0.40	ERM
18	C3F8	Off	0.20	0.88	− 0.68	Delayed surgery: MacON at VA check, MacOFF at surgery date.
19	C2F6	On	1.30	2.10	− 0.80	Post-Operative Corneal Graft haze with CMO
20	SF6	On	0.20	0.50	− 0.30	PCO
21	SF6	On	− 0.10	0.60	− 0.70	Cataract, ERM
22	C3F8	Off	0.20	0.50	− 0.30	Delayed surgery: MacON at VA check, MacOFF at surgery date.
23	C2F6	On	0.30	0.60	− 0.30	Cataract
24	SF6	Off	0.30	0.60	− 0.30	Uveitis
25	SF6	On	0.10	0.60	− 0.50	ERM
26	C3F8	On	− 0.10	2.40	− 2.50	IOL Dislocation
27	SF6	On	0.50	1.10	− 0.60	RVO
28	SF6	Off	1.00	1.30	− 0.30	Cataract
29	C3F8	On	0.30	0.70	− 0.40	Cataract
30	C3F8	Off	0.30	0.80	− 0.50	Cataract
31	SF6	On	0.00	0.50	− 0.50	Cataract
32	SF6	On	0.20	0.90	− 0.70	Cataract
33	C2F6	On	0.20	0.50	− 0.30	Cataract
34	C3F8	On	0.00	0.60	− 0.60	Cataract
35	SF6	On	0.20	0.80	− 0.60	Macular Fold
36	SO1000	Off	1.00	1.60	− 0.60	wAMD
37	SO1000	On	0.50	1.30	− 0.80	Post-operative Macular hole
38	SO1000	Off	2.40	3.00	− 0.60	Suprachoroidal Haemorrhage
39	Densiron	Off	0.00	0.50	− 0.50	Pre-operative advanced glaucoma, with rapid progression post-operatively.
40	SO5000	On	0.80	2.40	− 1.60	Viral Retinitis with posterior pole involvement post operatively
41	Densiron	On	0.50	0.80	− 0.30	Retained heavy liquid, chronic CMO, post operative uveitis
42	SO1000	On	0.20	0.50	− 0.30	CMO, ERM, PCO

*wAMD*:Wet Age Related Macular Degeneration, *PCO*:Posterior Capsular Opacification, *ERM*:Epiretinal membrane, *IOL*:Intraocular Lens,*MacON*:Macula-on retinal detachment, *MacOFF*:Macula-off retinal detachment, *CMO*:Cystoid Macular Oedema

All risk factors had univariate analysis for unexplained and all causes of visual loss (Additional file 1: Table S1). We found that GRT, the use of PFCL, PVR C, retinectomy, and SO use all contributed to increased risk of unexplained visual loss. To explore whether duration of SO tamponade contributed to unexplained visual loss, we reconducted the logistic regression with it as a covariate and limited the analysis to SO patients only (n = 61) as there were no instances of Densiron related unexplained visual loss. We did not find SO tamponade duration to be associated with unexplained visual loss (Odds Ratio 1.00 [95% confidence interval 0.98 to 1.03], p = 0.569).

**Fig. 2:**
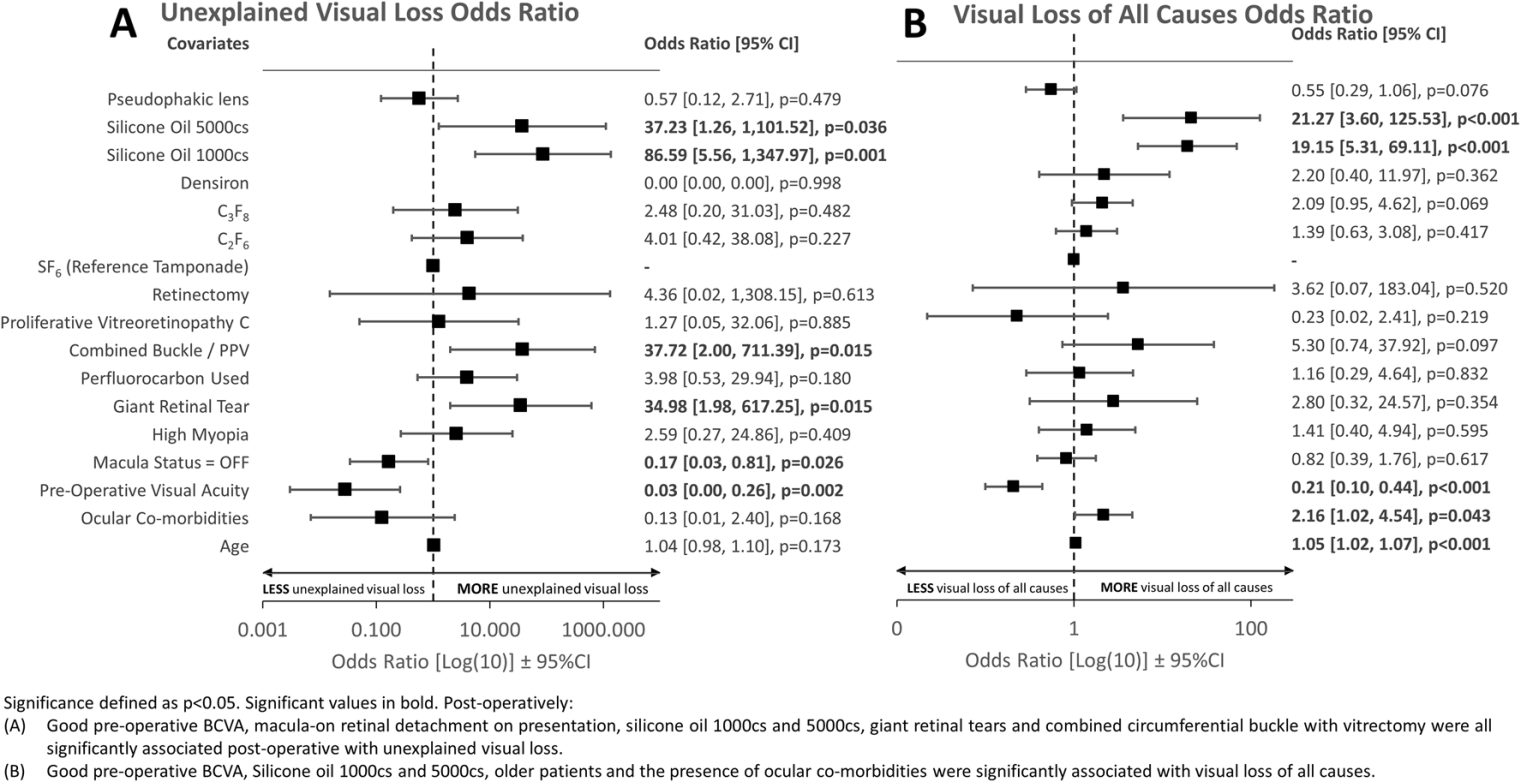
Forest plot of multivariable binary logistic regression model following primary retinal detachment repair for **A** Unexplained visual loss, **B** Visual Loss of all causes

A risk adjusted multivariable model is presented in Fig. 2, which shows a forest plot of two multivariable binary logistic regression models for unexplained visual loss and all causes of visual loss (Figure A and B, respectively). Post-operatively, SO 1000cs (p = 0.001) and 5000cs (p = 0.036) tamponade, GRTs and combined PPV and scleral buckle (p = 0.015 for both) were all significantly associated with unexplained visual loss (Fig. 2A); whereas, any SO tamponade, older age (p < 0.001 for all) and the presence of ocular co-morbidities (p = 0.043) were significantly associated with visual loss of all causes (Fig. 2B). Low pre-operative VA was significantly less likely to lead to both visual loss and unexplained visual loss following surgery (p < 0.001 and p = 0.002, respectively) (Fig. 2), whereas, macula-on RRD (p = 0.026) was associated with unexplained visual loss only (Fig. 2A).

We further dichotomised the eyes into HSO and SO (Table 3) and found these groups did not significantly differ for any baseline and operative findings, except for the intraoperative use of PFCL, that was significantly more common in SO group (p = 0.038). This separate analysis of eyes confirmed that the SO was more likely to be associated with unexplained visual loss than the Densiron, but not visual loss of all causes (Table 3). Despite our risk adjusted model, we noted several subgroups are inherently at higher risk of unexplained visual loss, including GRT, combined buckle/PPV and the primary retinectomy groups. As such, we repeated the logistic regression excluding these specific subgroups (remaining n = 989) and found that SO 1000cs remained a significant independent variable for unexplained visual loss relative SF_6_ (p < 0.001) and SO 5000cs trending toward significance (p = 0.078) (Additional file 2: Table S2).

**Table 3:** Baseline clinical characteristics and outcomes of primary retinal detachments by oil type

	Total	Densiron	Silicone Oil	p Value
Total		33	61	-
Age (years, IQR)	62 (53 to 72)	61 (55 to 74)	62 (51 to 69)	0.158
Gender (% Male)	71 (75.5%)	26 (78.8%)	45 (73.8%)	0.626
Laterality (% Right)	48 (51.1%)	17 (51.5%)	31 (50.8%)	1.000
High Myope (% Yes)	6 (6.4%)	3 (9.1%)	3 (4.9%)	0.662
Ocular Co-morbidities	34 (36.2%)	15 (45.5%)	19 (31.1%)	0.184
Preoperative Lens				
Phakic	45 (57.7%)	18 (60.0%)	27 (56.3%)	0.816
Pseudophakic	33 (42.3%)	12 (40.0%)	21 (43.8%)	
Macula Status				
Off	67 (71.3%)	23 (69.7%)	44 (72.1%)	0.815
On	27 (28.7%)	10 (30.3%)	17 (27.9%)	
Giant Retinal Tear	6 (6.4%)	0 (0.0%)	6 (9.8%)	0.087
PVR C	13 (13.8%)	3 (9.1%)	10 (16.4%)	0.532
Retinectomy	3 (3.2%)	0 (0.0%)	3 (4.9%)	0.549
Perfluorocarbon	20 (21.3%)	3 (9.1%)	17 (27.9%)	**0.038**
Combined Buckle/PPV	5 (5.3%)	0 (0.0%)	5 (8.2%)	0.158
Presentation to Surgery (days)	2 (0 to 7)	2 (0 to 7)	2 (0 to 7)	0.707
Postoperative Lens				
Phakic	9 (9.6%)	3 (9.1%)	6 (9.8%)	1.000
Pseudophakic	85 (90.4%)	30 (90.9%)	55 (90.2%)	
Duration of oil (days)	147 (91 to 215)	84 (68 to 124)	186 (133 to 233)	**< 0.001**
Visual Loss	15 (16.0%)	2 (6.1%)	13 (21.3%)	0.076
Unexplained Visual Loss	8 (8.5%)	0 (0.0%)	8 (13.1%)	**0.047**

*GRT:Giant Retinal Tear, PVR:Proliferative Vitreoretinopathy*

*Age and days from review to operation days are reported as median (interquartile range) and Kruskal Wallis test used to compare continuous variables*

*Chi Squared test to compare more than two nominal groups*

*Statistical significance in bold*

## Discussion

We conducted the largest study comparing the rate of unexplained visual loss and vision loss of all causes following primary RRD repair with gas, conventional SO 1000cs and 5000cs, and Densiron 68. Unexplained visual loss following primary RRD repair with SO tamponade is an established complication, whose etiopathogenesis is still unknown, and the recovery of VA has been reported only in a minority of cases [3, 4, 11, 12, 13, 14, 15, 16, 17, 18 and 19]. Although in surgical practice, SO of different viscosities and brands are used; these nuisances were not reported in many studies on SO-related vision.

In this study, the rate of unexplained visual loss was significantly higher in SO groups (11.6% for SO 1000cs and 16.7% for SO 5000cs) than in gas group (0.8%) and Densiron 68 (0.0%). These results could appear to contradict a recent meta-analysis that reported that there was no significant difference in terms of unexplained vision loss between gas and SO [20]. However, this meta-analysis included eyes with different primary indication for surgery; whereas our results are consistent with previous studies analysing eyes treated for primary RRD repair that report significantly higher incidence of unexplained vision loss associated with SO relative to gas[3, 6]. The proportion of patients in our study with unexplained visual loss is in within the range described in previous studies (3.3–29.7%) [3, 4, 11, 12, 13, 14, 15, 16, 17 and 18]  This variability can be explained by the differences in definition of visual loss and the difficulty in quantifying change in VA in very low values of baseline VA, as is typical in SO tamponade cases. Additionally, SOs of different manufacturers can have varying level of impurities that might contribute to this phenomenon [1, 21]. Indeed, since biochemical toxicity has been suggested as potential pathogenetic mechanism and it has been demonstrated that the purity of SO can significantly impact on their potential effect on cell viability [22], reporting the type of SO is important in the analysis of data and comparison of different studies.

Although there was a difference in case complexity in the different tamponade groups, we used a multivariable risk adjusted model to compare unexplained visual loss between them. Through both univariate and multivariable regression analyses in a sample of 1,012 eyes, our study demonstrated that both SO 1000cs and SO 5000cs were significantly associated with unexplained visual loss post-operatively, along with pre-operative macula-on status, GRT and combined scleral buckle. These findings supported the previously reported association between GRTs and unexplained visual loss. Indeed, in the largest case series to date on unexplained visual loss following ROSO including 421 consecutive eyes that underwent ROSO over a 2-year period, Moya et al. reported a 3.3% overall incidence of unexplained visual loss with 50% observed in macula-on GRTs detachments [13]. As the SO group had higher proportion of patients with GRT, despite including GRT as an independent risk factor in our regression model, this may have been a source of bias. As such, we repeated our logistic regression model, excluding high risk patients that had primary retinectomy, GRTs or comb buckle/PPV who were all in higher proportions in the SO groups. Despite this, SO remained a significant independent risk factor for unexplained visual loss (Additional file 2: Table S2).

Regarding the association between SO and unexplained vision loss, several mechanisms have been proposed as causative factors of retinal damage following SO use. Lo et al. divided these mechanisms into mechanical stress of the oil and the potential SO’s toxic effects on the retinal pigment epithelium [11]. Knorr et al. described thinning of the outer plexiform layer, vacuolization of the photoreceptor layer, and oil vacuole penetration into all layers of the retina in enucleated eyes [23]. Christensen et al. demonstrated a significant thinning of inner retinal layers in eyes with SO-treated eyes compared to gas-treated eyes [6], suggesting neuronal cell loss in the macular area as a potential cause of the visual loss [4]. It has also been suggested that SO-related visual loss could be induced by a greater exposure of the macula to the light during ROSO due to the optical effect associated with the SO under the surgical microscope illumination [24]. However, this mechanism does not explain the discrepancy in visual loss between Densiron and SO. There have additionally been several reports that suggest reduced superior, relative to inferior, radial peripapillary capillary vessel density with SO tamponade [24, 25, 26]. Due to the heavier-than-water density of Densiron, this observation may be expected to be isolated to SO.

SO viscosity and duration of SO tamponade did not correlate with the proportion of unexplained visual loss. The role of SO retention time in this complication is still controversial. Roca et al. retrospectively reviewed 324 eyes after ROSO and found the duration of SO tamponade to be a significant risk factor for unexplained visual loss [16]. However, their results are confounded by the inclusion of multiple pathologies and by the absence of a multivariable analysis for the conclusive assessment of the influence of the several risk factors identified in the univariate analysis [16]. For instance, eyes with more complex RD may have had longer SO tamponade and, thus, be inherently more likely to experience unexplained visual loss. In contrast, other series reported no association between unexplained vision loss and duration of SO tamponade [13]. Additionally, Dubroux et al., found no difference in retinal structure due to the length of SO tamponade [27].

Interestingly, no significant association has yet been reported between unexplained vision loss and Densiron despite SO 5000cs being a majority constituent. We report no cases of unexplained visual loss in this subgroup. So far, only Lee et al. reported one patient experiencing unexplained visual loss out of 32 patients following removal of HSO [7]. This is consistent with the results of a recent case series of 75 eyes treated with temporary or indefinite Densiron tamponade, in which no case of unexplained vision loss was documented [19].

Prior literature has alluded to the difficulty of attributing unexplained visual loss to either the SO itself or the “impurities” contained [28]. It has been previously highlighted that different SO can vary significantly in composition and, in particular, low molecular weight components content, and that the toxicity profile should be assessed and referred to a certain compound rather than the chemical group [29]. Our data along with the variability of the reported rate of unexplained vision loss might suggest a role of the specific SO used (with a certain composition) instead of the entire class of SO itself. However, further studies would be required to investigate the definitive cause(s) of unexplained vision loss and the potential influence of SO composition on this complication. Although SO and HSO show a mostly overlapping spectrum of complications, it has been demonstrated that there are some differences between the two classes of compounds, as HSO showed an increased propensity to cause intraocular inflammation and intraocular pressure elevation.[30] The reason for this difference in unexplained vision loss rate after SO and HSO tamponade is still unclear, as well as it remains unknown if the pathogenetic mechanisms suggested for SO-related complications [24], apply to the same degree to Densiron68 or additional pathogenetic mechanisms are involved [2, 19, 30].

To assess if different factors could have been involved in the different rate of unexplained vision loss in SO and HSO groups, we examine these groups separately. The two groups resulted to be homogeneous for the baseline variable but differed significantly for the duration of tamponade (longer in SO group) and the intraoperative use of PFCL (higher in SO group). As mentioned above, SO duration did not result to be a factor influencing the rate of unexplained vision loss. With regard to PFCL, although we found significant association with unexplained visual loss on univariate analysis, this finding was not confirmed on multivariable analysis. A recent ex vivo experimental study suggested that the sequential use of multiple intraocular medical devices can impact in retinal cell viability, in particular if the removal is not complete [31]. However, whether this might have a role in the onset of unexplained vision loss needs further investigation.

We acknowledge that this study has some limitations, including its retrospective nature and lack of case randomisation. However, due to the limited use of HSO in the current surgical practice, obtaining large sample size with prospective studies can be difficult as demonstrated by the underpowered size and premature completion of the multicentre prospective randomised control HSO study [32]. On the other hand, a retrospective analysis allowed us to obtain a large sample with adequate size to perform subgroups’ analyses. This enabled us to produce the first and largest series reporting on the relationship between the use of different tamponades, including conventional SO of different viscosity and HSO, and unexplained visual loss as well as on other causes of visual loss following ROSO. Although all patients with unexplained visual loss had at least an OCT scan, additional investigations such as microperimetry or electrophysiology tests were not performed. Additionally, we did not report on the single surgery anatomical success rate (or following ROSO) as anatomical success was a prerequisite to determine the difference in final VA between each tamponade. Therefore, differences in primary success rate may counteract those of unexplained vision loss between densiron and SO tamponade. However, within our cohort of patients, we did not find differences in primary success [33].

## Conclusion

Unexplained visual loss following ROSO is a well-known complication of SO tampoande. Through a large sample size and a robust statistical analysis, this study supported the association between unexplained vision loss and conventional SO of different viscosities (both 1000cs and 5000cs), whilst demonstrated a lack of significant association between Densiron and post-operative unexplained visual loss with no significant difference to the gas tamponade group. Duration of SO tamponade was not found to be a factor influencing the onset of this complication. Further laboratory studies are required to investigate the pathogenetic mechanisms responsible of unexplained vision loss and the difference between SO and HSO, as well as prospective multicentre studies would be important to report more accurately on this complication.

## Supplementary Information

**Additional file 1: Table S1.** Univariate Model for Unexplained Visual Loss following primary retinaldetachment repair. Data are reported as median (interquartile range). MannWhitney U was used to compare continuous data (age, and visual acuity). Fisher-exact test was otherwise used to compare nominal groups. Statistical significance in bold.

**Additional file 2: Table S2.** Multivariable binary logistic regression model following primary retinaldetachment repair for Unexplainedvisual loss, VisualLoss of all causes.  

## Data Availability

Data are available upon reasonable request.
