# Supplementary material for: Unexplained visual loss in retinal detachment repair: comparing gas, silicone oil and heavy silicone oil by multivariable regression
Source: Int J Retina Vitreous. 2023 Apr 29;9:30. doi: 10.1186/s40942-023-00466-9 (PMC10148464; doi:10.1186/s40942-023-00466-9)
Supplement: Supplementary file 1 — Additional file 1: Table S1. Univariate Model for Unexplained Visual Loss following primary retinaldetachment repair. Data are reported as median (interquartile range). MannWhitney U was used to compare continuous data (age, and visual acuity). Fisher-exact test was otherwise used to compare nominal groups. Statistical significance in bold. [file 40942_2023_466_MOESM1_ESM.docx]

|  | **Unexplained Visual Loss** | | **p Value** | **Visual Loss of all causes** | | **p Value** |
| --- | --- | --- | --- | --- | --- | --- |
| **Variables** | **No = 997 (98.5%)** | **Yes = 15 (1.5%)** |  | **No = 955 (94.4%)** | **Yes = 57 (5.6%)** |  |
| Age* | 59 (53 to 68) | 58 (52 to 71) | 0.803 | 59 (53 to 67) | 53 (55 to 71) | **0.040** |
| Ocular Co-morbidities |  |  |  |  |  |  |
| No | 847 (98.5%) | 13 (1.5%) | 0.603 | 817 (95.0%) | 43 (5.0%) | 0.054 |
| Yes | 150 (98.7%) | 2 (1.3%) |  | 138 (90.8%) | 14 (9.2%) |  |
| Pre-Operative Visual Acuity* | 0.50 (0.20 to 1.50) | 0.30 (0.20 to 0.50) | 0.170 | 0.50 (0.20 to 1.60) | 0.20 (0.20 to 0.50) | **<0.001** |
| Macula Status |  |  |  |  |  |  |
| Off | 489 (98.4%) | 8 (1.6%) | 0.472 | 477 (96.0%) | 20 (4.0%) | **0.040** |
| On | 508 (98.6%) | 7 (1.4%) |  | 478 (92.8%) | 37 (7.2%) |  |
| High Myopia |  |  |  |  |  |  |
| No | 937 (98.5%) | 14 (1.5%) | 0.609 | 897 (94.3%) | 54 (5.7%) | 1.000 |
| Yes | 60 (98.4%) | 1 (1.6%) |  | 58 (95.1%) | 3 (4.9%) |  |
| Giant Retinal Tear |  |  |  |  |  |  |
| No | 992 (98.7%) | 13 (1.3%) | **0.004** | 950 (94.5%) | 55 (5.5%) | 0.055 |
| Yes | 5 (71.4%) | 2 (28.6%) |  | 5 (71.4%) | 2 (28.6%) |  |
| Perfluorocarbon Used |  |  |  |  |  |  |
| No | 966 (99.0%) | 10 (1.0%) | **<0.001** | 925 (94.8%) | 51 (5.2%) | **0.013** |
| Yes | 31 (86.1%) | 5 (13.9%) |  | 30 (83.3%) | 6 (16.7%) |  |
| Combined Buckle / PPV |  |  |  |  |  |  |
| No | 985 (98.6%) | 14 (1.4%) | 0.177 | 944 (94.5%) | 55 (5.5%) | 0.163 |
| Yes | 12 (92.3%) | 1 (7.7%) |  | 11 (84.6%) | 2 (15.4%) |  |
| PVR C |  |  |  |  |  |  |
| No | 983 (98.7%) | 13 (1.3%) | **0.022** | 941 (94.5%) | 55 (5.5%) | 0.226 |
| Yes | 14 (87.5%) | 2 (12.5%) |  | 14 (87.5%) | 2 (12.5%) |  |
| Retinectomy |  |  |  |  |  |  |
| No | 995 (98.6%) | 14 (1.4%) | **0.044** | 953 (94.4%) | 56 (5.6%) | 0.160 |
| Yes | 2 (66.7%) | 1 (33.3%) |  | 2 (66.7%) | 1 (33.3%) |  |
| SF_6_ |  |  |  |  |  |  |
| No | 657 (97.9%) | 14 (2.1%) | **0.026** | 627 (93.4%) | 44 (6.6%) | 0.084 |
| Yes | 340 (99.7%) | 1 (0.3%) |  | 328 (96.2%) | 13 (3.8%) |  |
| C_2_F_6_ |  |  |  |  |  |  |
| No | 663 (98.4%) | 11 (1.6%) | 0.784 | 631 (93.6%) | 43 (6.4%) | 0.193 |
| Yes | 334 (98.8%) | 4 (1.2%) |  | 324 (95.9%) | 14 (4.1%) |  |
| C_3_F_8_ |  |  |  |  |  |  |
| No | 760 (98.3%) | 13 (1.7%) | 0.541 | 731 (94.6%) | 42 (5.4%) | 0.631 |
| Yes | 237 (99.2%) | 2 (0.8%) |  | 224 (93.7%) | 15 (6.3%) |  |
| Densiron |  |  |  |  |  |  |
| No | 964 (98.5%) | 15 (1.5%) | 1.000 | 924 (94.4%) | 55 (5.6%) | 0.709 |
| Yes | 33 (100.0%) | 0 (0.0%) |  | 31 (93.9%) | 2 (6.1%) |  |
| Silicone Oil 1000cs |  |  |  |  |  |  |
| No | 959 (99.0%) | 10 (1.0%) | **<0.001** | 921 (95.0%) | 48 (5.0%) | **<0.001** |
| Yes | 38 (88.4%) | 5 (11.6%) |  | 34 (79.1%) | 9 (20.9%) |  |
| Silicone Oil 5000cs |  |  |  |  |  |  |
| No | 982 (98.8%) | 12 (1.2%) | **0.002** | 941 (94.7%) | 53 (5.3%) | **0.015** |
| Yes | 15 (83.3%) | 3 (16.7%) |  | 14 (77.8%) | 4 (22.2%) |  |
| Post-operative Lens |  |  |  |  |  |  |
| Phakic | 465 (98.7%) | 6 (1.3%) | 0.795 | 445 (94.5%) | 26 (5.5%) | 1.000 |
| Pseudophakic | 532 (98.3%) | 9 (1.7%) |  | 510 (94.3%) | 31 (5.7%) |  |

**Supplementary Table 1: Univariate Model for Unexplained Visual Loss following primary retinal detachment repair**

*-Data are reported as median (interquartile range). Mann Whitney U was used to compare continuous data (age, and visual acuity). Fisher-exact test was otherwise used to compare nominal groups.*

*-Statistical significance in bold*
