# Supplementary material for: Unexplained visual loss in retinal detachment repair: comparing gas, silicone oil and heavy silicone oil by multivariable regression
Source: Int J Retina Vitreous. 2023 Apr 29;9:30. doi: 10.1186/s40942-023-00466-9 (PMC10148464; doi:10.1186/s40942-023-00466-9)
Supplement: Supplementary file 2 — Additional file 2: Table S2. Multivariable binary logistic regression model following primary retinaldetachment repair for Unexplainedvisual loss, VisualLoss of all causes. [file 40942_2023_466_MOESM2_ESM.docx]

Supplementary Table 2: Multivariate binary logistic regression model following primary retinal detachment repair for

1. Unexplained visual loss

| **Independent variable** | **B Coefficient** | **Odds Ratio (95% CI)** | **p Value** |
| --- | --- | --- | --- |
| Age | 0.055 | 1.046 (0.988 to 1.109) | 0.107 |
| Ocular Co-morbidities | -16.665 | 0.130 (0.007 to 2.453) | 0.995 |
| Pre-Operative Visual Acuity | -2.406 | 0.029 (0.003 to 0.264) | **0.031** |
| Macula Status (REF On) | -0.814 | 0.175 (0.035 to 0.863) | 0.376 |
| High Myopia | 0.664 | 2.779 (0.290 to 26.598) | 0.613 |
| Perfluorocarbon Used | 0.700 | 4.387 (0.569 to 33.830) | 0.554 |
| Proliferative Vitreoretinopathy C | 1.193 | 0.995 (0.037 to 27.040) | 0.526 |
| Tamponade Agent (REF SF_6_) |  |  |  |
| C_2_F_6_ | 1.645 | 5.180 (0.555 to 48.343) | 0.149 |
| C_3_F_8_ | 0.593 | 1.809 (0.109 to 29.904) | 0.679 |
| Densiron | -14.203 | -* | 0.998 |
| Silicone Oil 1000cs | 4.623 | 101.763 (6.659 to 1,555.223) | **<0.001** |
| Silicone Oil 5000cs | 3.618 | 37.264 (0.666 to 2,084.193) | 0.078 |
| Post-operative Lens (REF Pseudophakic) | -0.423 | 0.655 (0.127 to 3.377) | 0.613 |

1. Visual Loss of all causes.

| **Independent variable** | **B Coefficient** | **Odds Ratio (95% CI)** | **p Value** |
| --- | --- | --- | --- |
| Age | 0.045 | 1.047 (1.019 to 1.074) | **0.001** |
| Ocular Co-morbidities | 0.789 | 2.202 (1.044 to 4.647) | **0.038** |
| Pre-Operative Visual Acuity | -1.569 | 0.208 (0.099 to 0.436) | **<0.001** |
| Macula Status (REF On) | -0.192 | 0.825 (0.387 to 1.760) | 0.619 |
| High Myopia | 0.349 | 1.417 (0.403 to 4.985) | 0.587 |
| Perfluorocarbon Used | 0.155 | 1.168 (0.292 to 4.674) | 0.826 |
| Proliferative Vitreoretinopathy C | -1.507 | 0.222 (0.021 to 2.358) | 0.212 |
| Tamponade Agent (REF SF_6_) |  |  |  |
| C_2_F_6_ | 0.333 | 1.395 (0.629 to 3.096) | 0.413 |
| C_3_F_8_ | 0.751 | 2.119 (0.958 to 4.689) | 0.064 |
| Densiron | 0.837 | 2.311 (0.423 to 12.615) | 0.334 |
| Silicone Oil 1000cs | 3.018 | 20.458 (5.631 to 74.332) | **<0.001** |
| Silicone Oil 5000cs | 3.119 | 22.626 (3.813 to 134.250) | **0.001** |
| Post-operative Lens (REF Pseudophakic) | -0.698 | 0.498 (0.258 to 0.961) | **0.038** |

Significance defined as p<0.05. Significant values in bold. Post-operatively:

1. Good pre-operative visual acuity and Silicone oil 1000 cs (relative to SF_6_) were significantly associated post-operative with unexplained visual loss.
2. Silicone oil 1000cs and 5000cs, older patients, good pre-operative visual acuity and the presence of ocular co-morbidities were significantly associated with visual loss of all causes.
